# Dietary Lecithin Supplementation Can Improve the Quality of the M. *Longissimus thoracis*

**DOI:** 10.3390/ani5040405

**Published:** 2015-11-25

**Authors:** Darryl N. D’Souza, Bronwyn L. Blake, Ian H. Williams, Bruce P. Mullan, David W. Pethick, Frank R. Dunshea

**Affiliations:** 1 Australian Pork Limited, Level 2, 2 Brisbane Ave, Barton, Australian Capital Territory 2600, Australia; E-Mail: darryl.dsouza@australianpork.com.au; 2 Department of Agriculture and Food Western Australia, South Perth, Western Australia 6151, Australia; E-Mails: bronwyn.blake@agric.wa.gov.au (B.L.B.); bruce.mullan@agric.wa.gov.au (B.P.M.); 3 School of Animal Biology, The University of Western Australia, Crawley, Western Australia 6009, Australia; E-Mail: ian.williams@uwa.edu.au; 4 School of Veterinary and Life Sciences, Murdoch University, Murdoch, Western Australia 6150, Australia; E-Mail: D.Pethick@murdoch.edu.au; 5 Faculty of Veterinary and Agricultural Sciences, The University of Melbourne, Parkville, Victoria 3010, Australia

**Keywords:** lecithin, pork quality, texture, compression

## Abstract

**Simple Summary:**

Meat tenderness and texture can be influenced by the connective tissue content. Dietary lecithin offers a means of improving fat digestibility of pigs and reducing the connective tissue of pork. This feeding study confirmed that dietary lecithin decreased the chewiness and improved the fatty acid composition of pork without impacting on growth performance of pigs. Therefore, dietary lecithin supplementation has the potential to improve the quality attributes of pork.

**Abstract:**

Forty crossbred (Large White × Landrace × Duroc) female pigs (16.4 kg ± 0.94 kg) were used to investigate the effect of dietary lecithin supplementation on growth performance and pork quality. Pigs were randomly allocated to a commercial diet containing either 0, 3, 15 or 75 g lecithin/kg of feed during the grower and finisher growth phase. Pork from pigs consuming the diets containing 15 g and 75 g lecithin/kg had lower hardness (*P* < 0.001) and chewiness (*P* < 0.01) values compared to the controls. Dietary lecithin supplementation at 75 g/kg significantly increased (*P* < 0.05) the linoleic acid and reduced (*P* < 0.05) the myristic acid levels of pork compared to the control and the 3 g/kg and 15 g/kg lecithin supplemented treatments. Pigs fed the 75 g/kg lecithin supplemented diet had lower plasma cholesterol (*P* < 0.05) at slaughter compared to pigs fed the control diet and the 3 g/kg and 15 g/kg lecithin supplemented treatments. These data indicate that dietary lecithin supplementation has the potential to improve the quality attributes of pork from female pigs.

## 1. Introduction

Tenderness and texture are considered by consumers as perhaps the most important organoleptic or sensory attributes of pork and can be influenced by connective tissue, myofibril and the sarcoplasmic protein components of meat [1,2]. Collagen, a major component of intramuscular connective tissue is a key factor affecting the tenderness of meat [3]. The stability of collagen is dependent on the cross-linking of collagen fibrils [4] and the degree of cross-linking of collagen is dependent on the amount of hydroxyproline present in collagen [5]. The enzyme responsible for the hydroxylation of proline is prolyl-4-hydroxylase [6]. Therefore, the amount of collagen, the extent of collagen cross-linking and the types of collagen can all influence meat texture [7] and ultimately pork quality.

It has been hypothesized that polyenylphosphatidylcholine (PPC), a phospholipid present in lecithin extracted from soya bean, may decrease the amount of collagen or the extent of cross-linking of collagen fibrils. For example, PPC inhibits prolyl-4-hydroxylase resulting in reduced collagen fibril cross-linking and stimulates collagenase causing collagen breakdown in the liver of nonhuman primates [8,9]. In pigs, dietary lecithin (3 mg/kg) has been shown to decrease the compression characteristic of pork [10,11], possibly through decreasing the amount of collagen or the extent of collagen cross-linking [11]. Additionally, dietary lecithin supplementation reduces cholesterol absorption and low density lipoprotein cholesterol in humans [12] and it is possible that it may have a similar effect in pigs. Since alterations in pork lipid content through dietary lecithin supplementation may offer an important marketing opportunity for the pork industry [13,14], the aim of the present experiment was to determine the appropriate lecithin dose to improve the quality attributes of the m. *Longissimus thoracis.* This muscle was chosen as it has a relatively high consumer eating quality failure rate [15].

## 2. Experimental Section

### 2.1. Animal, Feeding and Experimental Design

The protocol used in this experiment conformed to all Animal Experimentation Ethics Committee (Activity No. 04SP044) regulations concerning the health and care of experimental animals.

Forty crossbred (Large White × Landrace × Duroc) female pigs (16.4 kg ± 0.94 kg) were stratified on a liveweight basis into 10 blocks and within each block randomly allocated to one of the following: (i) Control (pigs fed a commercial grower and finisher phase diet); (ii) Lecithin 3—3 g Lecithin (Ultralec soya bean lecithin, ADM Australia Pty. Ltd.)/kg of feed supplementation during the grower and finisher growth phase; (iii) Lecithin 15—15 g Lecithin/kg feed during the grower and finisher growth phase; or (iv) Lecithin 75—75 g Lecithin/kg of feed supplementation during the grower and finisher growth phase. Pigs were individually housed and had *ad libitum* access to feed, and water via nipple drinkers throughout the study. The composition of the grower and finisher phase diets are presented in Table 1 (all diets were formulated using the AUSPIG computer model [16]. The basal diets were prepared as a mash and the lecithin powder was mixed thoroughly with the basal diet to produce the experimental diets.

**Table 1:** Ingredient composition (%) of the basal grower and finisher pig diets ^1^.

Ingrendent	Grower Diet	Finisher Diet
Barley	10.0	67.1
Wheat	70.0	
Lupins	6.26	25.0
Soybean meal	1.00	
Blood meal	2.50	
Meat & bone meal	6.36	6.30
Fishmeal	2.00	
Canola oil	1.27	1.30
Lysine	0.189	0.027
Methionine	0.047	0.031
Threonine	0.056	
Mineral vitamin premix	0.070	0.070
Choline	0.040	0.040
Salt	0.200	0.100
Limestone		0.057
**Estimated Composition ^2^**		
Digestible energy (MJ/kg)	14.3	13.0
Protein%	18.7	18.2
Available lysine (g/MJ DE)	0.60	0.50
Total fat%	4.14	4.57

^1^ The basal diets were prepared as a mash and the lecithin powder (Ultralec, ADM) was mixed thoroughly with the basal diet to produce the experimental diets. ^2^ Estimated from the composition of the individual ingredients.

### 2.2. Slaughter

The pigs were transported to a commercial abattoir and slaughtered according to standard commercial procedures at 100 kg (±3.1 kg) liveweight. The pigs were stunned using a carbon dioxide dip-lift stunner set at 85% CO_2_ for 2 min (Butina, Denmark). Exsanguination, scalding, dehairing and evisceration were performed according to standard commercial procedures. The carcasses (head, flare fat, fore and hind trotters removed) were split before entering the chiller (5 °C to −1 °C cycle, air speed 5 m/s).

### 2.3. Growth Performance and Carcass Quality Assessment

The liveweight and feed intake of all the pigs were measured, and average daily gain (ADG) and feed conversion ratio (FCR) determined on a weekly basis. At the time of exsanguination, blood samples were collected from all pigs to assess plasma cholesterol concentrations [17]. After slaughter, the carcass weight and backfat depth at the P2 site (65 mm from the midline at the last rib) was determined on the hot carcass at 45 min post-slaughter. Back fat depth was measured using the Hennessy Grading Probe 4 (HGP 4).

### 2.4. Meat Quality Assessment

Twenty-four hours post-slaughter the m. *Longissimus thoracis* of the left-hand side of the carcass was removed for the assessment of pork quality. Muscle pH was determined using a portable pH/temperature meter (Jenco Electronic Ltd, Model 6009) fitted with a polypropylene spear-type gel electrode (Ionode IJ42S, Brisbane, QLD) and a temperature probe. Surface exudate was measured using the filter paper absorption method [18]. Surface lightness (L), redness (a) and yellowness (b) was measured with a Minolta Chromameter CR-400, using D65 illumination, a 2° observer, and an 8 mm aperture in the measuring head, standardised to a white tile. Meanwhile, m. *Longissimus thoracis* samples for texture analysis were vacuum packed and left to age for five days at 4 °C and then −20 °C while those for fatty analyses were frozen at −80 °C as described by Bouten *et al.* (1972) [19].

### 2.5. Pork Texture Assessment

The cooking procedure was adopted from the method described by Bouten *et al.* (1971) of [19]. The loin muscle was cut to a 70 ± 5 g cube (40 × 40 × 40 mm). The cube was weighed then cooked in water bath at 70 °C for 35 min. After removal from the water bath, the samples were allowed to cool in ice cold water for 20 min, patted dry to remove excess moisture and re-weighed before being refrigerated overnight. The weight loss during cooking was calculated as a percentage of weight loss before and after cooking.

Assessment of the cooked meat texture was determined using Warner–Bratzler shear force and compression analyses as described by Bouten *et al.* (1972) [20]. For shear force, the sample was cut into six rectangular strips of 1 cm^2^ cross section, parallel to the muscle fibres. Shear force blade (V-shaped) was fitted to the LF Plus machine (Lloyd Instruments Ltd., Fereham Hants, UK) and the crosshead speed was set at 300 mm/min and a 1 kilo newton (kN) load cell was used. The mean of the peak shear force was used as an estimate of tenderness. For compression analysis, the sample was cut into six cross-section samples (1 cm thick) with the fibres lying perpendicular on the face of the largest area. A flat-ended plunger with 0.63 cm surface diameter was fitted to the LF Plus machine. Firstly, the plunger was driven vertically at about 80% through the sample. The peak force required for the first compression was measured and this is defined as hardness. Secondly, the plunger was withdrawn and then returned to the same damaged area to measure the work done in repeating the first action. Cohesiveness is defined as the ratio of the work done during the second compression and that done during the first compression. Chewiness is defined as the product of hardness and cohesiveness [20].

### 2.6. Fatty Acid Analysis

Fatty acid analysis was conducted using a capillary gas chromatography (GC) [21] and cholesterol level analysis using a cholesterol assay kit (Sigma Aldrich, NSW, Australia). The details on sample preparation, extraction procedure and fatty acid quantification were described by Ponnampalam *et al.* (2010) [22]. The muscle samples were freeze-dried and a homogeneous sample of 0.5 g of ground material was used for the determination of fatty acid composition. One hundred µL of nonadecanoic acid methyl ester (C19: 0; Sigma Aldrich, NSW, Australia) was added to the muscle samples as an internal standard dissolved in chloroform (10 mg C19: 0/mL CHCl_3_). The sample solutions were hydrolysed using 0.7 mL of 10N potassium hydroxide (KOH) in water and 5.3 mL of methanol to form free fatty acids. After mixing well with vortex, the sample solutions were incubated at 55 °C for 1.5 h with vigorous mixing at 20-min intervals and then cooled to room temperature using tap water. Upon cooling, the sample solutions were mixed with 0.6 mL of 24N sulfuric acid in water. After mixing, the incubation and cooling process were preceded. After cooling the sample solutions to room temperature, the fatty acid methyl ester (FAME) in the sample solutions were separated with 1 mL of hexane solvent by mixing for 5 min and centrifuging at 2000× *g* for 10 min. Two hundred mL of hexane containing FAME was collected into a GC vial and fatty acid fractions were quantified by capillary GC (HP INNOWAX 60 m × 0.25 mm, 0.5 micron; Agilent J & W Scientific, Santa Clara, CA, USA). The fatty acid peaks were identified using a reference standard (Supelco C4–C24 mix; Sigma Aldrich, NSW, Australia), which was run in each batch.

### 2.7. Statistical Analysis

Analysis of variance (ANOVA) was used to analyse the main effects of diet and their effect on growth performance, carcass quality and pork quality using the using the Genstat program (Version 15, release 15.2.0.8821). When there were significant effects or trends (*P* < 0.10), the data were analysed for linear and quadratic effects as well as regression analyses to determine whether there were dose-dependent responses.

## 3. Results

There was no effect (*P* > 0.05) of dietary lecithin on average daily gain (ADG), average daily feed intake (ADFI) and feed conversion ratio (FCR) (Table 2). As a consequence there was no effect of dietary lecithin on final live weight, carcase weight or P2 back fat (Table 2).

While there was no effect of dietary lecithin on shear force and cohesiveness, there were dose-dependent linear and quadratic decreases in both hardness and chewiness of the *Longissimus thoracis* muscle with the responses for both reaching plateau at 15 g/kg of dietary lecithin (Table 3). These responses were confirmed by regression analyses of where it was shown that there was an exponential decrease in hardness (Y = 4.89−0.357 × log (X + 1) where Y = hardness in kg and X = dose of lecithin in g/kg, R = −0.38, *P* = 0.011) and chewiness (Y = 1.97−0.153 × log (X + 1) where Y = chewiness in kg and X = dose of lecithin in g/kg, R = −0.36, *P* = 0.015) in response to increasing doses of dietary lecithin that approached an asymptote at around 15 g/kg of dietary lecithin. There was no effect of dietary lecithin on ultimate pH, relative lightness (L *), redness (a *) and yellowness (b *), surface exudates and cook loss (Table 3).

**Table 2:** The effect of dietary lecithin supplementation on growth performance of female pigs.

	Dietary Lecithin (g/kg)					
	0	3	15	75	LSD ^1^	*P*-value
ADG (g/day)	869	848	853	849	95.0	0.94
ADFI (kg/day)	2.82	2.86	2.80	2.70	0.160	0.43
FCR	3.26	3.38	3.39	3.15	0.260	0.45
Final weight (kg)	100.7	101.7	101.5	102.1	3.1	0.85
Carcase weight (kg)	69.0	70.4	70.5	71.4	2.4	0.43
P2 (mm)	13.8	13.5	14.2	14.6	2.0	0.80

^1^ Least significant difference at *P* = 0.05. ADG, average daily gain; ADFI, average daily feed intake; FCR, feed conversion ratio.

**Table 3:** The effect of dietary lecithin supplementation on objective pork quality for the *Longissimus thoracis* muscle from female pigs.

	Dietary Lecithin (g/kg)					
	0	3	15	75	LSD ^1^	*P*-value
Shear force (kg)	5.80	6.06	5.76	6.01	1.24	0.95
Hardness (kg) ^2^	4.93	4.65	4.32	4.28	0.346	<0.001
Chewiness (kg) ^3^	1.99	1.88	1.73	1.72	0.163	0.002
Cohesiveness	0.404	0.404	0.398	0.402	0.016	0.82
pH (24 h)	5.45	5.47	5.50	5.46	0.099	0.81
Lightness (L *)	50.4	50.3	49.5	51.9	3.50	0.55
Redness (a *)	6.70	6.84	5.50	6.46	1.23	0.14
Yellowness (b *)	3.85	4.03	3.06	4.2	1.15	0.22
Surface exudate (mg)	68.4	59.4	54.0	68.7	23.5	0.52
Cook loss (%)	30.6	30.7	30.5	31.0	2.24	0.97

^1^ Least significant difference at *P* = 0.05; ^2^ Linear (*P* = 0.003) and quadratic (*P* = 0.006) effects; ^3^ Linear (*P* = 0.006) and quadratic (*P* = 0.010) effects.

There were linear effects of dietary lecithin on muscle linoleic (*P* = 0.01) and myristic (*P* < 0.05) acids such that dietary 75 g/kg lecithin increased (*P* < 0.05) the linoleic acid and reduced (*P* < 0.05) the myristic acid levels of pork compared to the control and the 3 g/kg and 15 g/kg lecithin supplemented treatments (Table 4). Regression analyses confirmed linear responses in both linoleic (Y = 15.9 + 0.0621 × X where Y = linoleic acid in % of total fatty acids and X = dose of lecithin in g/kg, *R* = 0.39, *P* = 0.010) and myristic (Y = 1.81−0.00375 × X where Y = myristic acid in % of total fatty acids and X = dose of lecithin in g/kg, *R* = −0.33, *P* = 0.068) acids although these responses were largely because of the impact of the highest dose of lecithin. The ratio of polyunsaturated fatty acids (PUFA): saturated fatty acids (SFA) tended (*P* = 0.058) to increase linearly with increasing dietary lecithin content although regression analyses didn’t confirm this relationship. There was a linear effect (*P* < 0.05) of dietary lecithin on plasma cholesterol such that pigs fed the 75 g/kg lecithin supplemented diet had lower plasma cholesterol (*P* < 0.05) at slaughter compared to pigs fed the control diet (Table 4). These response were confirmed by regression analyses where it was shown that there was an exponential decrease in plasma cholesterol (Y = 2.63−0.288 × log (X + 1) where Y = plasma cholesterol in mM and X = dose of lecithin in g/kg, *R* = −0.34, *P* = 0.030) in response to increasing doses of dietary lecithin which didn’t appear to be maximised within the dose range investigated.

**Table 4:** Fatty acid composition (%), intramuscular fat (%) of the *Longissimus thoracis* muscle and plasma cholesterol concentrations in female pigs fed diets supplemented with lecithin.

	Dietary Lecithin (g/kg)					
	0	3	15	75	LSD ^1^	*P*-value
Myristic acid ^2^	1.8	1.7	1.9	1.5	0.26	0.041
Palmitic acid	23.3	22.3	22.0	23.1	1.21	0.12
Heptadecanoic acid	0.47	0.57	0.36	0.15	0.554	0.47
Stearic acid	11.1	11.2	11.1	11.4	0.72	0.78
Oleic acid	33.8	34.7	34.0	31.2	4.65	0.46
Linoleic acid ^3^	16.6	16.8	15.4	20.8	3.91	0.043
Eicosadienoic acid	4.1	4.0	3.3	3.4	1.24	0.45
PUFA:SFA ^4,5^	0.58	0.62	0.53	0.70	0.151	0.15
Intramuscular fat (%)	1.3	1.2	1.1	1.2	0.33	0.91
Plasma cholesterol (mM) ^6,7^	0.423 (2.65)	0.336 (2.17)	0.396 (2.49)	0.268 (1.85)	0.1132	0.045

^1^ Least significant difference at *P* = 0.05; ^2^ Linear (*P* = 0.020) effect; ^3^ Linear (*P* = 0.010) effect; ^4^ PUFA polyunsaturated fatty acid; SFA saturated fatty acids; ^5^ Linear (*P* = 0.058) trend; ^6^ Data were log-transformed before analyses. Back transformed values are in parentheses. ^7^ Linear (*P* = 0.018) effect.

## 4. Discussion

The major finding from the present study was that dietary lecithin supplementation caused dose-dependent decreases in chewiness and hardness values for the *m. Longissimus thoracis* of pigs. D’Souza *et al.* [10] reported that 3 g/kg dietary lecithin supplementation reduced hardness and chewiness values of the *m. Semitendinosus,* whilst in this experiment, the reduced hardness and chewiness values of the *m. Longissimus thoracis* were reported for the 15 g/kg and 75 g/kg lecithin treatments and not the 3 g/kg lecithin treatment group. The study by Akit *et al.* [11] found that lecithin at 4, 20 and 80 g/kg reduced cohesiveness and chewiness of the m. *Longissimus lumborum* with no statistical within lecithin dose effects. However, the hardness values for pork from pigs fed 4 g/kg were intermediate between controls and those fed 20 g/kg [11]. The difference in hardness values for the *m. Semitendinosus* and the *m. Longissimus thoracis* and *lumborum* may be indicative of the differences in muscle characteristics such as muscle bundle size and the size of the perimysium surrounding the muscle bundles. The m. *Semitendinosus* has smaller muscle bundles and a thinner perimysium surrounding the muscle bundles compared to the *m* *Longissimus thoracis* and *lumborum* [1] and this may explain the lower hardness values and also the lower dose of dietary lecithin required to reduce the compression characteristics of the m. *Semitendinosus* compared to the m. *Longissimus thoracis* and *lumborum.*

While dietary supplementation with lecithin at 15 and 75 g/kg resulted in significant decreases in chewiness and hardness, there was no difference in shear force values between the dietary lecithin supplementation and the control treatments. The shear force, hardness and chewiness values reported in this experiment were 15%–20% higher compared to those reported by Channon *et al.* [2] who suggested that pork with a shear force values >5 kg were perceived by trained consumers as being tough. The correlation between objective measures (compression and shear force) and sensory evaluation of eating quality attributes especially tenderness has shown to be extremely variable, especially at low shear forces [23]. In light of this, it is possible that the reduction in hardness and chewiness values observed in the present studies and those of D’Souza *et al* [10] and Akit *et al.* [11] were insufficient to decrease the shear force of pork considered tender (shear force < 5 kg) pork. In the only study where sensory studies were conducted on pork from pigs fed dietary lecithin, there was no effect of 8 g/kg dietary lecithin on shear force, juiciness or tenderness there was a decrease in pork that scored below average and therefore failed to satisfy consumers [24]. Also, the intention to repurchase was higher for pork from pigs supplemented with lecithin [24].

It has been suggested that polyenylphosphatidylcholine (PPC), a phospholipid, present in lecithin extracted from soya bean, may decrease the amount of collagen or the extent of cross-linking of collagen fibrils. Studies have shown that PPC inhibits prolyl-4-hydroxylase resulting in reduced collagen fibril cross-linking and stimulates collagenase causing collagen breakdown in the liver of nonhuman primates [8,9]. The active component of PPC may be dilinoleoylphosphatidylcholine (DLPC) which has been shown to have an anti-fibrogenic effects in the liver of alcoholics [25] by stimulating the enzyme collagenase. Studies by Li *et al.* [25] demonstrated that addition of polyunsaturated lecithin (10 μmol/L), which contains DLPC, increased collagenase activity in the liver by 100%. This increase in collagenase aided in the prevention of excess collagen accumulation by offsetting increased collagen production typical in patients suffering from liver fibrosis. Dilinoleoylphosphatidylcholine has also been shown to decrease the incorporation of proline into secreted collagen in hepatic stellate cells extracted from rats [26]. If DLPC has the same effect in the muscles of pigs it will decrease the thermal stability of the molecules. The content of both proline and hydroxyproline are important in the thermal stability of collagen molecules. The ring structure of proline assists in maintenance of the helical structure of the molecule. Areas lacking in proline cause the helix to unwind and become unstable. A reduction in proline may also lead to a decrease in hydroxyproline. This will subsequently lead to a decrease in the thermal stability of the molecules and an increase in the solubility of the fibres. The antifibrogenic effects of DLPC have, up to now, only been studied in liver cells damaged by alcohol. It is possible that the effects outlined above may also occur in muscle cells. Collagenase activity, hydroxyproline content, types of cross-links or collagen solubility was not tested in this experiment so the authors cannot further elucidate the mode of action of lecithin. However, Akit *et al.* [24] found that muscle hydroxyproline content was reduced in pigs consuming diets supplemented with lecithin indicating that the collagen content was also reduced.

These data also indicate that dietary lecithin supplementation (75 g/kg) reduced the myristic acid (14:0) and increased the linoleic acid (18:2) content of the *m* *Longissimus thoracis* which is consistent with the findings of Akit *et al.* [27] who fed up to 80 g/kg of dietary lecithin. The ratio of PUFA:SFA tended to increase with increasing dietary lecithin in the present study which again is consistent with Akit *et al.* [27] who found the PUFA:SFA ratio increased from 0.46 to 0.73 when the dietary lecithin content was increased from 0 to 80 g/kg. In both studies the highest PUFA:SFA ratio was achieved within the range of 75–80 g/kg which may be cost prohibitive to be used for just modification of the fat content. Consumer surveys have indicated that consumers are willing to pay more for cholesterol or fat reduced pork [13,14] so it is possible that dietary lecithin, may offer some possibility to value add pork, however, these need to be further investigated. Although, Akit *et al.* [27] found that intramuscular fat was decreased (from 2.1% to 1.5%) by dietary lecithin, there was no effect in the present study. However, intramuscular fat was very low (*ca.* 1.3%) here and it may have been difficult to further reduce intramuscular fat.

Although muscle cholesterol wasn’t measured in the present study, plasma cholesterol did decrease with increasing dietary lecithin with the exception being the 15 g/kg lecithin supplementation treatment. The authors cannot speculate as to why the pigs supplemented with 15 g/kg lecithin did not have lower cholesterol levels. Akit *et al.* [27] found that plasma cholesterol tended to be decreased in pigs supplemented with lecithin although this was not statistically significant. Human studies have found that lecithin supplementation significantly reduced cholesterol plasma [12]. Lecithin in humans has been shown to reduce cholesterol and increases the PUFA:SFA in both serum and erythrocytes [12]. The most likely rationale for this is that lecithin, an emulsifying agent, improves the digestibility of triglycerides. Therefore, in plant-based diets that are higher in PUFA than SFA, a supplement of lecithin should lead to a higher absorption and deposition of PUFA like linoleic acid. By contrast, when lecithin combines with cholesterol the resulting micelle is much larger than one formed with bile salts alone, possibly resulting in a decreased absorption of cholesterol from the gut.

In the present study there was no effect of dietary lecithin on growth performance. There have been very few studies conducted with dietary lecithin in grower/finisher pigs and effects on ADG and FCR have been variable. Studies from our laboratories have failed to observe any effect on ADG, final liveweight or FCR [10,11,24], although in one study there was in increase in dressing rate [11]. In contrast, Kim *et al.* [28] reported that dietary lecithin improved ADG and feed efficiency and suggested that the improved feed efficiency was due to a more efficient dietary tallow digestibility given the emulsifying properties of lecithin. Dietary lecithin has been reported to enhance utilisation of dietary fat, particularly in young pigs [29,30]. However, dietary lecithin doesn’t appear to improve fat digestibility in grower [31] or finisher [32] pigs. Importantly, there were no reductions in growth performance with dietary lecithin supplementation so if this strategy was used to improve the quality attributes of pork there should be no negative production consequences.

## 5. Conclusions

Dietary lecithin supplementation reduced the chewiness and hardness of pork, and has the potential to improve the tenderness of pork. The effects of dietary lecithin on pork chewiness and hardness appear to be maximized at 15 g/kg, whereas the effects on plasma cholesterol and muscle fatty acids require 75 g/kg dietary lecithin. However, the use of lecithin supplementation in pig diets and its subsequent effect on collagen cross-linking, shear force, sensory pork quality and the fatty acid composition of pork need to be further investigated.
